# Shuci method of electroacupuncture improves self-perception and quality of life in patients with knee osteoarthritis: a multicenter randomized controlled trial

**DOI:** 10.3389/fmed.2026.1864999

**Published:** 2026-08-26

**Authors:** Yiming Chen, Hongyu Xie, Min Ye, Jin Wang, Danni Zhang, Aihong Yuan

**Affiliations:** 1 The First Affiliated Hospital of Anhui University of Chinese Medicine, Hefei, Anhui, China; 2 Key Laboratory of Xin'an Medicine, Ministry of Education, Hefei, Anhui, China

**Keywords:** acupuncture, CGI-I, knee osteoarthritis, randomized controlled trial, Shuci

## Abstract

**Background:**

Knee osteoarthritis (KOA) is a common chronic disease characterized by degenerative damage and disability of the knee joint. Current treatment options for KOA have considerable limitations. The Shuci theory for treating KOA within the traditional Chinese acupuncture medical system has evolved into a specific modern therapy: the Shuci method of electroacupuncture. Although it has been widely applied at the level of clinical experience, high-level evidence remains lacking.

**Methods:**

In this multicenter, patient- and assessor-blinded, superiority randomized controlled trial, 110 patients with KOA were randomly assigned in a 1:1 ratio to an electroacupuncture (EA) group or a sham EA (SEA) group, receiving treatment three times per week for two consecutive weeks. Acupuncture was performed following the Shuci theory. Patients were assessed at baseline and after completing treatment. The primary outcome was response as defined by the Clinical Global Impressions scale - Improvement. Secondary outcomes included the Visual Analogue Scale (VAS), pressure pain threshold (PPT), and Knee Injury and Osteoarthritis Outcome Score (KOOS). In accordance with the Intention-to-Treat principle, all randomized patients formed the primary analysis set. The primary outcome was analyzed using a Z-test for proportions. Secondary outcomes were analyzed using a mixed-effects model.

**Results:**

After treatment, the response rate was 76.4% (42 of 55) in the EA group and 27.3% (15 of 55) in the SEA group, with a between-group difference of 49.1% (95% CI: 32.8, 65.4%; *p* < 0.001). The mixed-effects model analysis revealed that the EA group showed significantly greater improvements than the SEA group in VAS [Difference (95% CI): −2.1 (−2.6, −1.6); *p* < 0.001], PPT [20.1 (14.9, 25.3); *p* < 0.001], KOOS-pain [23.3 (18.3, 28.3); *p* < 0.001], KOOS-Symptoms [16.8 (10.8, 22.8); *p* < 0.001], KOOS-Activities of daily living [7.2 (0.1, 14.3); *p* = 0.046], and KOOS-Knee-related quality of life [7.6 (3.0, 12.2); *p* = 0.001].

**Conclusion:**

We preliminarily confirmed the temporary efficacy of the Shuci method of electroacupuncture for KOA.

**Clinical trial registration:**

https://www.chictr.org.cn, ChiCTR2300078135.

## Graphical abstract

**Figure:**
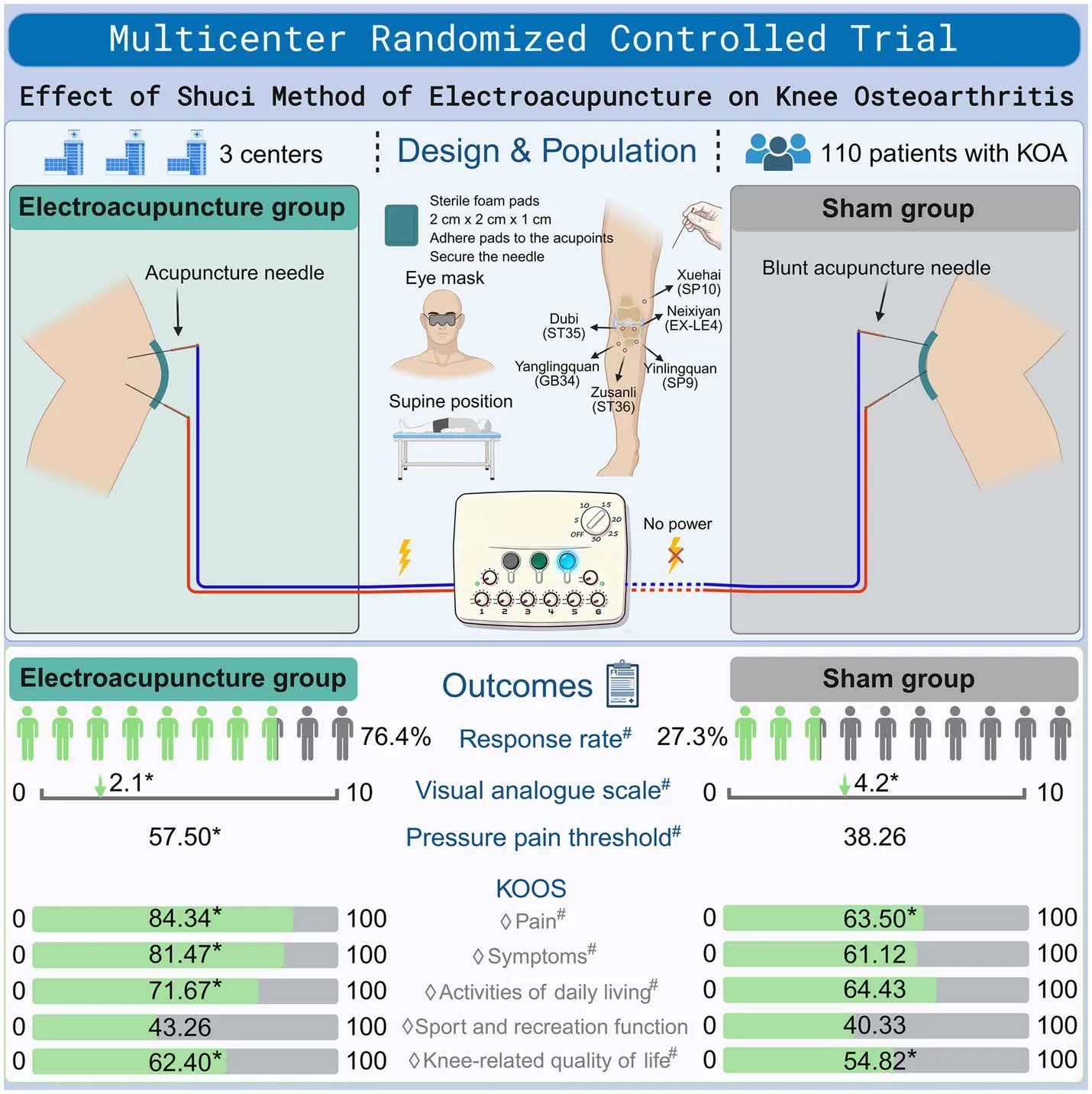
*Compared with own baseline, *p* < 0.05. ^#^Comparison of differences between two groups after treatment, *p* < 0.05.

## Introduction

Knee osteoarthritis (KOA) is a common chronic joint disease worldwide, characterized by degenerative damage and disability of the knee joint (1). The Global Burden of Diseases study reported that in 2021, the global prevalent cases of KOA were 370 million, incident cases were 30 million, and disability-adjusted life years totaled 12 million (2). From 2021 to 2045, the global age-standardized prevalence rate of KOA will increase by 7.8% (from 4,294 to 4,630 per 100,000), and the age-standardized death rate will increase by 5.7% (from 137 to 145 per 100,000) (3). The high prevalence and high disability rate of KOA not only cause suffering to patients but also impose a heavy social burden.

Currently, KOA remains a disease with unclear etiology and complex pathogenesis. Factors such as mechanical load, inflammation, metabolism, and aging play key roles in the onset and progression of KOA (4). KOA pathology can involve the cartilage, bone, synovium, fascia, and muscles, leading to irreversible joint damage. The persistent and intractable pain of KOA is characterized by neuropathic pain, involving mechanisms of dual sensitization of both the peripheral and central systems induced by inflammation and structural changes (5–7). Due to these complex mechanisms, treatment options for KOA have substantial limitations. The mainstream treatments for moderate-to-advanced KOA are pharmacotherapy and surgery. The first-line drugs, nonsteroidal anti-inflammatory drugs, can only alleviate inflammatory pain in KOA but carry the risk of multiple adverse effects (8). Knee surgery is costly, associated with slow recovery, and carries high risks (9). Most importantly, they cannot stably control the persistent pain and improve quality of life (QoL).

Acupuncture is a traditional therapeutic strategy that has been widely recognized internationally (10). A systematic review of international clinical practice guidelines for chronic musculoskeletal pain conditions found that 60% of the guidelines recommend the acupuncture (11). The ancient Chinese medical classic *Huangdi Neijing*, with a history of over 2,000 years, records an acupuncture theory: “The Shuci refers to direct needling into the joint until deep contact with the bone, can be used to treat Gubi (the ancient Chinese term for KOA).” This therapy has been used continuously to the present day. Moreover, modern acupuncturists have combined acupuncture with controlled, regular electrical stimulation to form electroacupuncture (EA), which further enhances the therapeutic effect (12). A randomized controlled trial (RCT) of different acupuncture techniques for KOA (13) showed that EA was the most effective among the five techniques. In summary, the Shuci method of electroacupuncture has been commonly used for KOA in China at the level of clinical experience.

However, there remains a lack of high-level clinical evidence for the Shuci method of electroacupuncture in KOA. Therefore, we conducted this multicenter, patient- and assessor-blind, superiority RCT. Using multiple self-perception and QoL measures, we evaluated the efficacy and safety of the Shuci method of electroacupuncture for KOA. We believe this trial will clearly demonstrate the performance of acupuncture in KOA and provide evidence-based support for optimizing comprehensive treatment strategies for KOA. Graphical abstract shows a graphical presentation of this trial.

## Methods

### Study design

**Figure 1:**
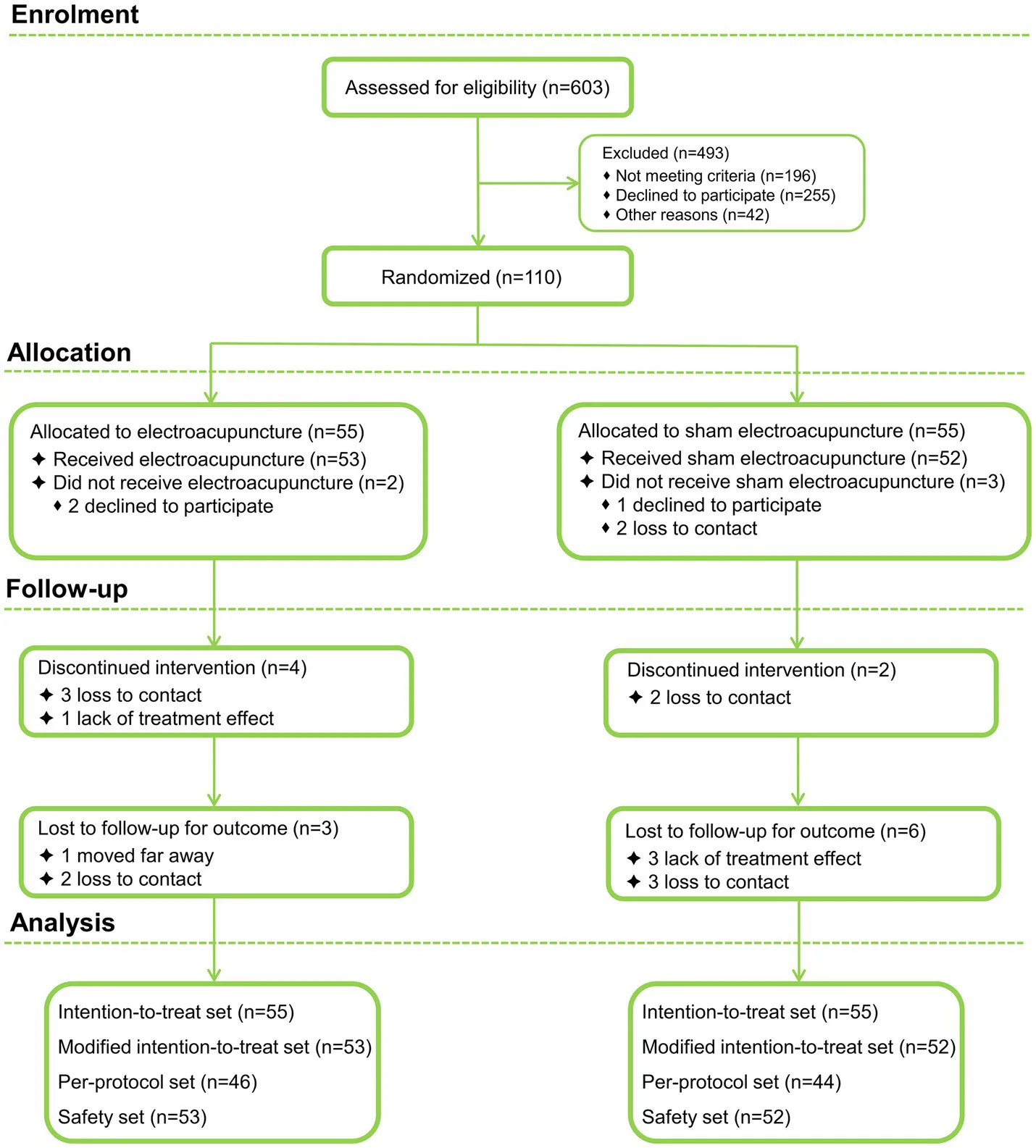
The consolidated standards of reporting trials flow diagram.

This trial adopted a multicenter, patient- and assessor-blind, superiority, randomized controlled design. Eligible KOA patients were recruited from three centers and allocated at a 1:1 ratio to the EA group or the SEA group using stratified block randomization. Participants were assessed at baseline and after completing the 2-week treatment. Figure 1 presents the Consolidated Standards of Reporting Trials flow diagram.

### Participants and criteria

Participants in this trial were inpatients and outpatients from the First Affiliated Hospital of Anhui University of Chinese Medicine, Anhui Provincial Hospital, and Bozhou Hospital of Chinese Medicine. Eligibility of potential participants was assessed by professional recruiters with extensive clinical experience using strict inclusion and exclusion criteria.

All diseases were diagnosed by senior specialist physicians at the time of recruitment after inquiring about and reviewing the patient’s symptoms, signs, laboratory tests, and medical history. Diagnoses also referred to the patient’s previous medical records from any healthcare facility. These senior specialist physicians had more than 10 years of clinical experience in their specialty and were not involved in any other aspect of the trial.

#### Diagnosis criteria

The diagnosis of KOA referenced the American College of Rheumatology’s Classification of Osteoarthritis of the Knee (14) and the 2024 Chinese guidelines for diagnosis and treatment of osteoarthritis (15). Specifically, the diagnosis can be made when criterion ① plus any two of the other criteria are met:

(1) Recurrent knee pain within the last month;
(2) Age ≥ 50 years;
(3) Morning knee stiffness ≤ 30 min;
(4) Bony crepitus with knee joint movement;
(5) X-ray showed narrowing of the knee joint space, subchondral bone sclerosis or cystic changes, and osteophyte formation at the joint margins.

#### Inclusion criteria

(1) Met the diagnostic criteria;
(2) Aged between 50 and 75 years;
(3) Persistent knee pain over the past 3 months;
(4) Visual Analogue Scale (VAS) ≥ 4.0;
(5) No acupuncture treatment in the past 3 months;
(6) Informed consent and good compliance.

#### Exclusion criteria

(1) Presence of other musculoskeletal disorders;
(2) Presence of psychiatric, cerebrovascular, or neurological diseases;
(3) Use of medications affecting mental state, cognition, pain perception, or brain function within the past 3 months;
(4) Pregnancy, lactation, or plans for pregnancy in the near future;
(5) Lower limb deformities or disabilities affecting normal walking;
(6) Had undergone knee joint injection and surgery within the past 6 months;
(7) Current or planned participation in other clinical trials.

#### Withdrawal criteria

(1) Inability to cooperate or lost contact to various reasons;
(2) Severe adverse reactions, complications, or disease progression;
(3) Poor compliance or failure to adhere to the treatment protocol;
(4) Receipt of additional acupuncture, knee joint injections, or surgery during the study period;
(5) Breaking of the blind.

### Randomization, allocation concealment, and blinding

The stratified block randomization method was used, with stratification factors being center and disease duration. Within each stratum, the block length was set to 4 or 6, with an allocation ratio of 1:1. A statistician generated the randomization sequence using a central randomization system. The statistician submitted the participants’ stratification data to the system, which automatically assigned the groups, denoted by the letters A or B.

Due to the operational nature of acupuncture, only the acupuncturists were unavoidably aware of the participants’ letter assignments and corresponding treatment. However, the acupuncturists did not participate in recruitment, allocation, assessment, or analysis. Assessments were conducted by assessors. Finally, a statistician performed the statistical analysis. The recruiters, acupuncturists, assessors, and statistician all worked independently and did not communicate with each other.

During treatment, participants in the two groups were assigned to different locations, thereby preventing communication that could lead to unblinding. After completing the treatment, all participants were asked to guess which type of acupuncture they had received, and Bang’s Blinding Index (BBI) (16) was used to determine the success of blinding.

### Sample size calculation

Considering the superiority design of this study, we estimated the sample size based on the response rates defined by CGI-I ≤ 2. The calculation was performed using the R package TrialSize. According to previous studies on acupuncture for KOA (12, 17), the response rate ranged from 45.0 to 60.3% in trial group and from 30.8 to 47.3% in control group. A clinically meaningful difference in response rate was considered to be at least 20%. Using a two-sided chi-square test with *α* = 0.05 and 80% power, the calculated sample size was at least 94 participants (47 per group). Accounting for a 15% dropout rate, the final sample size was set at 110 participants (55 per group).

### Interventions

#### Electroacupuncture group

Participants lay supine, wore an eye mask, and the local skin was disinfected. The acupoints Neixiyan (EX-LE4), Dubi (ST35), Yanglingquan (GB34), Yinlingquan (SP9), Zusanli (ST36), and Xuehai (SP10) were selected (Supplementary Table S1). We adhered to the Shuci theory from traditional Chinese medicine (TCM) system for treating KOA, which requires needling toward the knee joint to a depth reaching the bone surface. Patients with bilateral KOA received bilateral acupuncture (12 acupoints), while those with unilateral KOA received unilateral acupuncture (6 acupoints). A sterile foam pad (2 cm × 2 cm × 1 cm) was affixed to the skin. Needles (0.35 mm × 50 mm) were inserted through the pad into the acupoints until the needle tip reached the bone surface. Twirling, lifting and thrusting manipulations were then performed to achieve deqi. Subsequently, an electroacupuncture device was connected to the needles at Neixiyan and Dubi. Parameters: dense-sparse wave, frequency 2/100 Hz, intensity 2 mA. Needles were retained for 30 min before removal. Treatment was administered three times per week for two consecutive weeks. Acetaminophen was given on demand, except within 48 h before outcome assessment.

#### Sham electroacupuncture group

SEA, a placebo control, involved the use of a specially designed blunt-tip needle that penetrated the foam pad to reach the skin surface without piercing the skin. The needle remained upright and maintained a certain pressure on the skin with the support of the pad. The tactile sensation induced by this skin pressure leads patients to mistakenly perceive that they have received acupuncture. The electroacupuncture device was also connected but not powered. Patients could hear the timing sound of the mechanical timer knob of the device. To maximize blinding, all patients were informed that the current intensity might be below the threshold of human perception. Other procedures for SEA were the same as those for EA. The similarities and differences between the two groups are presented in Table 1.

**Table 1:** Similarities and differences between the groups.

Characteristics	**Electroacupuncture**	**Sham electroacupuncture**
Preparation	Supine position; Eye mask; Sterilized	Identical
True or sham acupoints?	True	Identical
Number of acupoints	6	Identical
Types of needles?	Acupuncture needle	Blunt acupuncture needle
Depth	Bone surface	Skin surface
De qi	Yes	No
Electroacupuncture device	Connect; Power	Connect; No power
The parameters of the device	dense-and-sparse wave; 2/100 Hz; 2 mA	Identical
Needle retention, min	30	Identical
Acupuncture strategies	Three times / week for 2 weeks	Identical
Acetaminophen	As needed	Identical

### Outcomes

At baseline and after 2 weeks of treatment, an assessor conducted face-to-face interviews with each participant and supervised the completion of the outcome questionnaires. Supplementary Table S2 presents the participant timeline of tasks.

#### Primary outcome

The primary outcome of this trial was response. Response was defined as the Clinical Global Impressions scale - Improvement (CGI-I) ≤ 2 (Supplementary Figure S1). The CGI-I is a 7-point self-report scale published by the U. S. Department of Health, Education, and Welfare in 1976 (18). It is primarily used to quantify a patient’s overall self-perception of changes in illness and QoL after treatment. Specifically: 1, very much improved; 2, much improved; 3, minimally improved; 4, no change; 5, minimally worse; 6, much worse; 7, very much worse.

#### Secondary outcomes

(1) VAS (19): It is one of the most common methods for quantifying pain, in which patients are asked to mark their perceived level of pain on a 10-cm straight line, with 0 representing no pain and 10 representing the most severe pain.
(2) Pressure pain threshold (PPT) (20): The minimum pressure applied at which the participant first began to feel pain was measured using an algometer. This is an objective indicator of mechanical pain sensitivity and directly reflects the degree of pain sensitization.
(3) Knee Injury and Osteoarthritis Outcome Score (KOOS) (21): A self-reported functional and QoL scale specifically designed for patients with knee injury and osteoarthritis, comprising five subdimensions: Pain, Symptoms, Activities of Daily Living (ADL), Sport and Recreation Function (SRF), and Knee-related Quality of Life (KQL). This scale comprehensively evaluates knee status and function. Each subdimension is scored from 0 to 100, where a score of 100 indicates completely normal.

#### Safety assessment

Based on their potential association with acupuncture, adverse events (AEs) were classified as acupuncture-related or unrelated. AEs were counted according to the type of event rather than the frequency within the same participant. Different types of AEs occurring in the same participant were recorded as separate AEs. The same type of AE occurring multiple times in one participant was recorded as a single AE.

### Statistical analysis

In accordance with the Intention-to-Treat (ITT) principle, all patients who underwent randomization formed the primary analysis set, i.e., ITT Set. This principle includes participants in the analysis according to their originally assigned groups, regardless of whether they completed the trial as planned. Under the missing at random assumption, multiple imputation was used to impute missing data. The imputation model included the following variables to enhance the plausibility and precision: group, center, age, sex, Body Mass Index (BMI), KOA duration, Kellgren & Lawrence (KL) grade, Patients’ Expectancy Scale of Acupuncture (PESA), and baseline values of all outcomes. A total of 50 imputed datasets were generated, followed by systematic diagnostic procedures to assess imputation quality. Convergence was evaluated by visual inspection of trace plots of the means and standard deviations (SDs) of imputed values across iterations. Plausibility of imputation was assessed by comparing density plots of observed and imputed values. Whether imputed values fell within the plausible range for each outcome was also evaluated. Rubin’s rules were used to combine the outcome means and their corresponding standard errors (SEs) across all plausible datasets.

At baseline, between-group comparisons were performed using the *t*-test or Mann–Whitney *U* test for continuous variables and the chi-square test for categorical variables.

For the primary outcome-response, the numbers of responders in the two groups were averaged across all datasets and rounded to the nearest integer for a more intuitive presentation of responder counts. Continuous variables were summarized as mean ± SD for baseline data derived from actual assessments and as pooled mean ± SE for post-treatment values derived from pooling after multiple imputation of missing data in mixed-effects model. Categorical variables were presented as counts and percentages.

The primary outcome, response rate, was compared between the two groups using a two-proportion *Z*-test, with the rate difference and its 95% confidence interval (CI) reported.

For secondary outcomes with repeated measurements (VAS, PPT, and KOOS), a mixed-effects model was used. The model included group, time (post-treatment vs. baseline), and their two-way interaction (treatment × time) as fixed effects. The primary inferential focus was on this interaction term, which directly tests whether the change from baseline to post-treatment differed significantly between the EA and SEA groups. Baseline values of the respective outcome were included as covariates to adjust for potential regression to the mean and baseline imbalance. Center and participant were included as random intercepts. Given that our trial involved only three centers, we treated center as a random effect to account for potential clustering, while acknowledging that the variance component for center would be estimated with limited precision. An unstructured covariance matrix was specified for the repeated measurements within each participant to allow for flexible correlations between baseline and post-treatment assessments. All adjusted between-group differences (least-squares mean differences) and their 95% CIs were derived from this model; they are not equivalent to the unadjusted descriptive mean changes from post-treatment to baseline.

No adjustment for multiple comparisons was applied to secondary outcomes, as these were exploratory in nature; however, results were interpreted with consideration of the potential for type I error. A two-sided significance level of *α* = 0.05 was used for all hypothesis tests.

Sensitivity analyses for the primary outcome were performed in Worst-case Scenario of ITT Set, Modified ITT Set, and Per-protocol Set to ensure the robustness of the results. The Worst-case Scenario of ITT Set assumed that all dropouts in EA group were unresponsive to treatment, while all dropouts in SEA group were responsive. The Modified ITT Set included all participants who completed at least one treatment session, excluding those who dropped out before the first treatment began. For the Modified ITT Set, multiple imputation was still used to handle missing data. The Per-protocol Set consisted of participants who completed all treatments and the final assessment, excluding those who deviated from the protocol.

Pre-specified subgroup analyses included: ① KL grade II or III; ② PESA >30 or ≤30. The interaction effects between these subgroup factors and acupuncture were analyzed. PESA (22): A tool specifically designed to assess patients’ expectations of acupuncture prior to treatment. It can be used to clarify the influence of psychological effects on the outcomes and to isolate the specific physiological effects of acupuncture. A higher score indicates a more positive attitude toward acupuncture.

All participants who received ≥1 treatment session were included in Safety Set, and the occurrence of adverse events was documented.

All statistical analyses were performed using SPSS Statistics v27.0 and R v3.6.

## Results

### Participants

Our recruitment commenced on 1 October 2024, was expected to conclude in September 2026, and was actually completed on 15 April 2026. The first screening occurred on 3 October 2024, the first informed consent was obtained on 6 October 2024, the first randomization was performed on 6 October 2024, the last assessment took place on 15 April 2026, and data were locked on 15 April 2026.

A total of 603 patients were screened, and 110 participants were enrolled and randomly assigned to the two groups (Figure 1). Thus, the ITT Set for the primary analysis consisted of 110 participants. Five participants dropped out before the first treatment, six did not complete the full treatment, and nine did not complete the outcome assessment. These lost participants contributed to missing data rates of 16.4% (9/55) and 20.0% (11/55) for the two groups, with an overall missing rate of 18.2% (20/110). All per-protocol participants completed full assessments with no missing data.

Consequently, the Modified ITT Set included 105 participants, the Per-protocol Set included 90 participants, and the Safety Set included 105 participants. Baseline characteristics of participants in ITT Set are presented in Table 2.

**Table 2:** Baseline characteristics of participants in Intention-to-Treat Set.

Characteristic	All (*n* = 110)	EA group (*n* = 55)	SEA group (*n* = 55)
Age, mean ± SD years	63.2 ± 7.2	62.6 ± 7.4	63.7 ± 7.1
Sex (female), *n* (%)	78 (70.9)	40 (72.7)	38 (69.1)
Race, *n* (%)			
Han	107 (97.3)	53 (96.4)	54 (98.2)
Minorities	–	–	–
BMI, mean ± SD kg/m^2^	26.0 ± 3.5	25.7 ± 3.3	26.2 ± 3.7
KOA duration, mean ± SD years	6.7 ± 5.4	6.5 ± 5.6	6.9 ± 5.2
KL grade, *n* (%)			
Grade II	68 (61.8)	33 (60.0)	35 (63.6)
Grade III	42 (38.2)	22 (40.0)	20 (36.4)
Affected knee (bilateral), *n* (%)	91 (82.7)	46 (83.6)	45 (81.8)
Past treatment, *n* (%)^a^			
Acupuncture	40 (36.4)	21 (38.2)	19 (34.5)
Physical therapy	53 (48.2)	26 (47.3)	27 (49.1)
Medication	75 (68.2)	36 (65.5)	39 (70.9)
Injections	7 (6.4)	4 (7.3)	3 (5.5)
Comorbidity, *n* (%)			
0	63 (57.3)	31 (56.4)	32 (58.2)
1	33 (30.0)	17 (30.9)	16 (29.1)
2	9 (8.2)	4 (7.3)	5 (9.1)
≥3	5 (4.5)	3 (5.5)	2 (3.6)
PESA, mean ± SD	33.26 ± 7.11	31.57 ± 5.42	34.95 ± 8.18
VAS, mean ± SD	5.3 ± 1.6	5.2 ± 1.8	5.4 ± 1.4
PPT, mean ± SD N	35.28 ± 15.34	34.87 ± 14.41	35.68 ± 16.34
KOOS, mean ± SD			
Pain	56.68 ± 15.06	55.42 ± 14.22	57.94 ± 15.88
Symptoms	59.02 ± 18.11	60.78 ± 19.80	57.26 ± 16.23
ADL	61.85 ± 23.34	63.10 ± 21.54	60.60 ± 25.15
SRF	39.34 ± 9.14	40.60 ± 8.36	38.08 ± 9.77
KQL	45.41 ± 11.41	43.15 ± 9.42	47.67 ± 12.80

^a^Defined as treatment previously sought for KOA.

### Efficacy

For the primary outcome (Table 3), the response rate after completing treatment was 76.4% (42 of 55 participants) in the EA group and 27.3% (15 of 55 participants) in the SEA group. The between-group difference between the EA and SEA groups was 49.1% (95% CI: 32.8, 65.4%; *p* < 0.001).

**Table 3:** Primary and secondary outcomes in Intention-to-Treat Set.

Outcomes	EA group (*n* = 55)	SEA group (*n* = 55)	Difference (95% CI)^a^	*p*
Response rate, *n* (%)	42 (76.4)	15 (27.3)	49.1 (32.8, 65.4)	<0.001
VAS				
Baseline, mean ± SD	5.2 ± 1.8	5.4 ± 1.4	–	–
Post-treatment, mean (SE)	2.1 (0.1)*	4.2 (0.2)*	−2.1 (−2.6, −1.6)	<0.001
PPT, N				
Baseline, mean ± SD	34.87 ± 14.41	35.68 ± 16.34	–	–
Post-treatment, mean (SE)	57.50 (1.74)*	38.26 (2.16)	20.1 (14.9, 25.3)	<0.001
KOOS				
Pain				
Baseline, mean ± SD	55.42 ± 14.22	57.94 ± 15.88	–	–
Post-treatment, mean (SE)	84.34 (1.60)*	63.50 (1.98)*	23.3 (18.3, 28.3)	<0.001
Symptoms				
Baseline, mean ± SD	60.78 ± 19.80	57.26 ± 16.23	–	–
Post-treatment, mean (SE)	81.47 (2.44)*	61.12 (2.84)	16.8 (10.8, 22.8)	<0.001
ADL				
Baseline, mean ± SD	63.10 ± 21.54	60.60 ± 25.15	–	–
Post-treatment, mean (SE)	71.67 (2.04)*	64.43 (1.95)	7.2 (0.1, 14.3)	0.046
SRF				
Baseline, mean ± SD	40.60 ± 8.36	38.08 ± 9.77	–	–
Post-treatment, mean (SE)	43.26 (1.20)	40.33 (1.41)	2.9 (−1.6, 7.4)	0.207
KQL				
Baseline, mean ± SD	43.15 ± 9.42	47.67 ± 12.80	–	–
Post-treatment, mean (SE)	62.40 (2.26)*	54.82 (2.53)*	7.6 (3.0, 12.2)	0.001

^a^Regarding the response rate, a *Z*-test for proportions was employed to report the difference between the groups, followed by the calculation of the rate difference and its 95% CI. For secondary outcomes, a mixed-effects model was utilized, accounting for the balance of centers, participants, and baseline characteristics. We primarily focused on the treatment × time interaction and its significance, specifically the adjusted between-group difference and *p*-value. The between-group differences are adjusted estimates derived from the mixed-effects model; they are not equivalent to the unadjusted descriptive mean changes from post-treatment to baseline. Data are presented as mean ± SD for baseline and mean (SE) for post-treatment values estimated from the mixed-effects model. *Compared with own baseline, *p* < 0.05.

For secondary outcomes, the mixed-effects model revealed that, compared with baseline, the EA group showed significant improvements in VAS, PPT, KOOS-pain, KOOS-Symptoms, KOOS-ADL, and KOOS-QOL after treatment (all *p* < 0.05). The SEA group showed significant improvements in VAS, KOOS-pain, and KOOS-QOL after treatment (all *p* < 0.05). Of note, the EA group showed significantly greater improvements than the SEA group in VAS [Difference (95% CI): −2.1 (−2.6, −1.6); *p* < 0.001], PPT [20.1 (14.9, 25.3); *p* < 0.001], KOOS-pain [23.3 (18.3, 28.3); *p* < 0.001], KOOS-Symptoms [16.8 (10.8, 22.8); *p* < 0.001], KOOS-ADL [7.2 (0.1, 14.3); *p* = 0.046], and KOOS-QOL [7.6 (3.0, 12.2); *p* = 0.001].

### Sensitivity analysis of the primary outcome in different sets

The difference in response rates between the two groups was re-examined in Worst-case Scenario of ITT Set, Modified ITT Set, and Per-protocol Set, respectively (Supplementary Table S3). The response rate in the EA group remained significantly higher than that in the SEA group in Worst-case Scenario of ITT Set [Difference (95% CI): 29.1 (11.3, 46.9); *p* = 0.002], Modified ITT Set [52.3 (36.1, 68.6); *p* < 0.001], and Per-protocol Set [57.6 (40.8, 74.4); *p* < 0.001].

### Subgroup analysis

Participants were divided into Grade II and Grade III subgroups according to KL grade, and into PESA >30 and PESA ≤30 subgroups according to PESA (Supplementary Table S4). The difference in response rates between the EA and SEA groups was analyzed again. The results showed that, across different subgroups, the differences in response rates between the EA and SEA groups remained similar to those described above. Neither the subgroup variable KL grade (*p* = 0.457) nor PESA (*p* = 0.551) showed an interaction with treatment.

### Safety

No severe AEs occurred in either the EA group or the SEA group (Table 4). Acupuncture-related AEs included subcutaneous hemorrhage and post-acupuncture pain. Since the SEA group did not insert needles into the body, no acupuncture-related AEs occurred, whereas the EA group experienced 5 cases and 3 cases, respectively. Acupuncture-unrelated AEs were rare in both groups. All AEs were mild and resolved spontaneously within 1 week.

**Table 4:** Adverse events.

Adverse events	EA group (*n* = 53)	SEA group (*n* = 52)
Total participants with AEs, n (%)	17 (32.1)	8 (15.4)
Total AEs	9	6
Severe AEs	0	0
Acupuncture-related AEs		
Subcutaneous hemorrhage	5	0
Post-acupuncture pain	3	0
Acupuncture-unrelated AEs		
Abdominal pain	0	1
Upper respiratory tract infection	1	0
Rash	1	0
Headache	2	2
Insomnia	0	1
Diarrhea	2	0
Constipation	1	1
Low back pain	3	2
Neck pain	2	2

Total participants with AEs, number of participants who experienced AEs. Total AEs, number of AE types. For each sub-item under acupuncture-related AEs and acupuncture-unrelated AEs, the number represents the count of participants who experienced that specific AE.

### Blinding

In the EA group, 41 participants guessed their treatment correctly, 5 guessed incorrectly, and 7 were uncertain, resulting in a BBI of 0.68. In the SEA group, 8 participants guessed correctly, 38 guessed incorrectly, and 6 were uncertain, resulting in a BBI of −0.58. It can be seen that most participants in both groups tended to believe they had received genuine treatment, indicating that blinding within each group was unsuccessful, yet the blinding of the trial was successful (Supplementary Table S5).

## Discussion

The core value of this multicenter RCT lies in systematically confirming the unique advantages of the Shuci method of electroacupuncture in improving self-perception and QoL in patients with KOA. The primary outcome of the trial was response defined by the CGI-I, which assesses the degree of improvement from the patient’s global self-perception, aligning with the actual clinical decision-making process (23). Our results showed that after treatment, the response rate in the EA group (76.4%) was significantly superior to that in the SEA group (27.3%). The nearly 50% difference in response rates between the two groups highlights the clinical importance of the Shuci method of electroacupuncture as an intensive intervention.

Additionally, the Shuci method of electroacupuncture demonstrated specific therapeutic effects beyond placebo in relieving pain (VAS), increasing pain threshold (PPT), and improving comprehensive knee function (multiple domains of KOOS). Furthermore, the recorded AEs indicated that the Shuci method of electroacupuncture is safe within a two-week period. These findings not only validate the vitality of the Shuci theory of TCM in modern clinical practice but also provide a solid evidence-based foundation for non-pharmacological and comprehensive treatment strategies for KOA.

The Shuci method vividly demonstrates the clinical features and advantages of acupuncture in “local targeting” (24, 25). Unlike previous acupuncture studies (13, 26) that focused only on the skin or muscle layers, the Shuci method emphasizes insertion from acupoints around the knee joint until the needle tip reaches the articular bone surface (24, 27, 28). The periosteum on the bone surface contains abundant nerve endings and vessels, making it an important site for articular pain transmission (29, 30). Delivering enhanced physical stimulation via EA may more effectively regulate local microcirculation and block pain signal transmission compared to superficial stimulation (31). This precise intervention targeting deep knee joint tissues (such as periosteum, cartilage, and joint capsule) may be extended to other chronic musculoskeletal disorders.

In the international discussion of acupuncture for KOA, the debate over its efficacy has never ceased. The study by Brian M. Berman et al. (32) demonstrated that acupuncture was partially superior to sham acupuncture. A multicenter three-arm RCT published in *Arthritis & Rheumatology* (12) showed that EA and acupuncture were both superior to SEA in improving pain, joint function, and QoL in KOA, and that EA had a stronger effect than acupuncture. The results of our trial are highly consistent with those studies. Nevertheless, the RCT by Rana S. Hinman et al. (33) indicated that in people over 50 years of age with knee pain, acupuncture was not superior to sham acupuncture in terms of knee pain and function. A possible reason for these seemingly contradictory results is the inherent difficulty in determining the appropriate acupuncture dose (34). As an operational treatment, variations in frequency, session number, needle type, depth, acupoint, direction, retention time, and manipulation technique represent differences in acupuncture dose (35, 36). More importantly, EA is also clearly an intensive dose. Differences in dose can significantly affect the efficacy of acupuncture, thereby potentially leading to negative outcomes.

In the subgroup analysis, we found that the superiority of the EA group remained stable regardless of pathological severity (KL grade) or level of PESA at baseline. This suggests that the effect of the Shuci method may primarily derives from its specific physical stimulation, rather than solely from population selection bias or psychological cueing. Notably, the SEA group also exhibited some degree of improvement in VAS and KOOS. This aligns with the common manifestation of placebo effects in acupuncture for chronic pain, whereby brain pathways are activated through expectancy effects and mild tactile stimulation (37). However, the substantial improvement in PPT observed in the EA group was not achieved in the SEA group, indicating that the Shuci method of electroacupuncture may possess significant biological specificity in modulating pain sensitization. A study has suggested that acupuncture directly acts on deep tissues, and may influence neuroplasticity by engaging ascending excitatory pathways and the descending pain modulation system (38). We need to recognize that the mechanism of acupuncture for KOA still holds great research potential.

Our strengths are as follows: ① This is a multicenter RCT validating the efficacy of the Shuci method of electroacupuncture in treating KOA, revitalizing a traditional medical theory in the modern era and providing high-level evidence for the clinical promotion. ② A variety of assessment tools, particularly CGI-I, were integrated to multidimensionally evaluate and analyze the global self-perception and QoL of patients, which aligns with real-world clinical decision-making processes.

The limitations of this trial are as follows: ① Due to the operational characteristics of acupuncture, we were unable to blind the acupuncturists. However, we made every effort to separate the roles of the acupuncturists. ② A treatment and observation period of only 2 weeks appears insufficient; further RCTs with long-term treatment and follow-up need to be planned. In addition, the use of acetaminophen also needs to be recorded. ③ The primary and most secondary outcomes were subjective and susceptible to potential bias inherent in self-reporting, although we performed blinding to the best of our ability and blinding was successful.

## Conclusion

This multicenter RCT has, to a certain extent, preliminarily demonstrated the temporary efficacy and safety of the Shuci method of electroacupuncture in improving self-perception and QoL in patients with KOA. We provide positive evidence for the application of traditional Chinese acupuncture theory within the modern evidence-based medicine framework, while also supporting the Shuci method of electroacupuncture as one of the options for comprehensive intervention in KOA.

## Data Availability

The raw data supporting the conclusions of this article will be made available by the authors, without undue reservation.
