# Supplementary material for: Shuci method of electroacupuncture improves self-perception and quality of life in patients with knee osteoarthritis: a multicenter randomized controlled trial
Source: Front Med (Lausanne). 2026 Aug 26;13:1864999. doi: 10.3389/fmed.2026.1864999 (PMC13553432; doi:10.3389/fmed.2026.1864999)
Supplement: Supplementary file 1 [file Data_Sheet_1.docx]

**Figure S1** The definition of response.

**Table S1** The location of the acupoints.

**Table S2** Schedule of tasks for participants.

**Table S3** Sensitivity Analysis of the Primary Outcome in different sets.

**Table S4** Subgroup analysis for Kellgren & Lawrence grade and patients' expectancy scale of acupuncture.

**Table S5** Blinding assessment.

**Modifications report**

**Figure S1** The definition of response.

**Table S1** The location of the acupoints.

| **Acupoints** | **Locations** |
| --- | --- |
| Neixiyan (EX-LE4) | On the knee, in the middle of the medial depression of the patellar ligament. |
| Dubi (ST35) | On the anterior aspect of the knee, in the depression lateral to the patellar ligament. |
| Yanglingquan (GB34) | On the fibular aspect of the leg, in the depression anterior and distal to the head of the fibula. |
| Yinlingquan (SP9) | On the tibial aspect of the leg, in the depression between the inferior border of the medial condyle of the tibia and the medial border of the tibia. |
| Zusanli (ST36) | On the anterior aspect of the leg, on the line connecting ST35 with ST41, 3 B-cun inferior to ST35. |
| Xuehai (SP10) | On the anteromedial aspect of the thigh, on the bulge of the vastus medialis muscle, 2 B-cun superior to the medial end of the base of the patella. |

**Table S2** Schedule of tasks for participants.

| **Study Period** | | | | |
| --- | --- | --- | --- | --- |
|  | **Enrollment** | **Allocation** | **Treatment** | |
|  | Week -1~0 | Week 0  (Baseline) | Week 1 | Week 2 |
| **Enrolment** |  |  |  |  |
| Eligibility screen | **x** |  |  |  |
| Informed consent | **x** |  |  |  |
| Demographic information | **x** |  |  |  |
| Medical history | **x** |  |  |  |
| Knee X-ray | **x** |  |  |  |
| Random Allocation |  | **x** |  |  |
| **Intervention** |  |  |  |  |
| Electroacupuncture |  |  | **x** | **x** |
| Sham electroacupuncture |  |  | **x** | **x** |
| **Assessments** |  |  |  |  |
| CGI-I |  |  |  | **x** |
| VAS |  | **x** |  | **x** |
| PPT |  | **x** |  | **x** |
| KOOS |  | **x** |  | **x** |
| PESA |  | **x** |  |  |
| **Safety** |  |  |  |  |
| Adverse events |  |  | **x** | **x** |
| **Blinding index** |  |  |  | **x** |

**Table S3** Sensitivity Analysis of the Primary Outcome in different sets.

| **Sets** | **EA group** | **SEA group** | **Difference (95% CI)** | ***p*** |
| --- | --- | --- | --- | --- |
| Worst-case Scenario of ITT Set | 38/55 (69.1) | 22/55 (40.0) | 29.1 (11.3, 46.9) | 0.002 |
| Modified ITT Set | 42/53 (79.2) | 14/52 (26.9) | 52.3 (36.1, 68.6) | < 0.001 |
| Per-protocol Set | 38/46 (82.6) | 11/44 (25.0) | 57.6 (40.8, 74.4) | < 0.001 |

Note: The Worst-case Scenario of ITT Set assumed that all dropouts in electroacupuncture group were unresponsive to treatment, while all dropouts in sham electroacupuncture group were responsive. The Modified ITT Set included all participants who completed at least one treatment session, excluding those who dropped out before the first treatment began. For the Modified ITT Set, multiple imputation was still used to handle missing data. The Per-protocol Set consisted of participants who completed all treatments and the final assessment, excluding those who deviated from the protocol.

**Table S4** Subgroup analysis for Kellgren & Lawrence grade and patients' expectancy scale of acupuncture.

| **Subgroup** | **EA group** | | **SEA group** | | **Difference (95% CI)** | ***p* for interaction** |
| --- | --- | --- | --- | --- | --- | --- |
|  | **n** | **Response (**%**)** | **n** | **response (**%**)** |  |  |
| **KL grade** |  |  |  |  |  |  |
| Grade Ⅱ | 33 | 28 (84.8) | 35 | 10 (28.6) | 56.2 (36.4, 76.0) | 0.457 |
| Grade Ⅲ | 22 | 14 (63.6) | 20 | 5 (25.0) | 38.6 (11.0, 66.2) |  |
| **PESA** |  |  |  |  |  |  |
| ＞30 | 37 | 31 (83.8) | 39 | 12 (30.8) | 53.0 (34.3, 71.7) | 0.551 |
| ≤ 30 | 18 | 11 (61.1) | 16 | 3 (18.8) | 42.3 (13.0, 71.6) |  |

**Table S5** Blinding assessment.

| **Guess, n (%)** | **EA group (n = 53)** | **SEA group (n = 52)** |
| --- | --- | --- |
| Acupuncture | 41 (77.4) | 38 (73.1) |
| Sham acupuncture | 5 (9.4) | 8 (15.4) |
| Unsure | 7 (13.2) | 6 (11.5) |
| Bang's Blinding Index (95% CI) | 0.68 (0.51, 0.85) | -0.58 (-0.78, -0.38) |

**Modifications report**

After registering the trial protocol on the platform, we amended some protocol content prior to the commencement of participant recruitment. These amendments have been approved. We report the modifications here:

1. The primary outcome was changed to CGI-I.

2. Secondary outcomes were changed to: VAS, PPT, KOOS.

3. Participants were allocated using a stratified block randomization method.

4. After recalculation, the sample size was set at 110 cases (55 per group).

5. The inclusion and exclusion criteria were modified as follows:

Inclusion criteria

① Met the diagnostic criteria;

② Aged between 50 and 75 years;

③ Persistent knee pain over the past three months;

④ Visual Analogue Scale (VAS) ≥ 4.0;

⑤ No acupuncture treatment in the past three months;

⑥ Informed consent and good compliance.

Exclusion criteria

① Presence of other musculoskeletal disorders;

② Presence of psychiatric, cerebrovascular, or neurological diseases;

③ Use of medications affecting mental state, cognition, pain perception, or brain function within the past three months;

④ Pregnancy, lactation, or plans for pregnancy in the near future;

⑤ Lower limb deformities or disabilities affecting normal walking;

⑥ Had undergone knee joint injection and surgery within the past six months;

⑦ Current or planned participation in other clinical trials.
